# 3D Echocardiographic Phenotyping of Left Ventricular Mechanics and Function With Contemporary Radiation Therapy

**DOI:** 10.1016/j.adro.2025.101786

**Published:** 2025-04-16

**Authors:** Matthew A. Barrett, Biniyam G. Demissei, Ray Hu, Amanda M. Smith, Gary Freedman, John Plastaras, Steven Feigenberg, Eva Berlin, Hari K. Narayan, Benedicte Lefebvre, Marielle Scherrer Crosbie, Michael Fradley, Joseph Carver, Jinbo Chen, Bonnie Ky

**Affiliations:** a Department of Medicine, Division of Cardiology, Perelman School of Medicine, University of Pennsylvania, Philadelphia, Pennsylvania; b Department of Radiation Oncology, Perelman School of Medicine, University of Pennsylvania, Philadelphia, Pennsylvania; c Division of Pediatric Cardiology, UC San Diego, San Diego, California; d Department of Biostatistics and Epidemiology, Perelman School of Medicine, University of Pennsylvania, Philadelphia, Pennsylvania

## Abstract

**Purpose:**

Our objective was to characterize the early changes in cardiac function after thoracic radiation therapy (RT) using 3D echocardiography.

**Methods and Materials:**

In a prospective longitudinal cohort study of 69 patients with breast cancer, lung cancer, or mediastinal lymphoma treated with chemotherapy and RT, clinical and 3D echocardiographic data were assessed before, immediately after, and 5 to 9 months after RT completion. 3D left ventricular ejection fraction, global circumferential strain, global longitudinal strain (GLS), average 3D strain, twist, and torsion were quantified. Associations among mean heart dose (MHD), V5, and V30 and early changes in echocardiography-derived measures of cardiac function were assessed with generalized estimating equations.

**Results:**

The median (quartile 1, quartile 3) estimates of MHD ranged from 1.2 Gy (1.0-1.9) in patients with breast cancer (*n* = 39), to 6.8 Gy (4.0-12.5) in patients with mediastinal lymphoma (*n* = 17), and 19.4 Gy (11.3-21.7) in patients with lung cancer (*n* = 13). There were no significant changes in 3D echocardiography measures in patients with breast cancer over time. However, in patients with lung cancer/lymphoma, there was a worsening in 3D left ventricular ejection fraction, GLS, and average 3D strain from pre-RT to RT completion (*P* < .05). This worsening in 3D GLS persisted at 5 to 9 months (*P* < .05). Across the entire cohort, MHD, V5, and V30 were not associated with changes in global 3D echocardiography-derived measures (*P* > .05).

**Conclusions:**

Early abnormalities in cardiac function as measured by 3D echocardiography can be detected following RT. Additional work is needed to define the determinants of changes in cardiac function with RT and long-term impact of early changes on clinical outcomes.

## Introduction

Advancements in cancer screening and treatment have led to increased survival in patients with cancer; however, thoracic radiation therapy (RT) has been associated with adverse cardiovascular effects.^1^, ^2^, ^3^, ^4^ Many of the seminal studies characterizing the adverse cardiac effects of RT have focused on late and clinically overt cardiac disease.^5^^,^^6^ The acute and subacute cardiac effects of contemporary RT are not well-defined.^7^, ^8^, ^9^, ^10^

Echocardiography represents a clinically important tool in the detection of cancer therapy cardiotoxicity. 2D left ventricular ejection fraction (LVEF) and deformation indices, which are limited by geometric assumption models, have been the standard evaluation of cancer therapy cardiotoxicity.^11^ 3D echocardiography overcomes these limitations through imaging of the heart in its complete dimensionality and has been shown to be more reproducible than corresponding 2D measurements.^12^^,^^13^ Additionally, there has been evidence to suggest that 3D LVEF is a better predictor of mortality compared to 2D LVEF.^14^ However, there has been a paucity of studies using 3D echocardiography applied to the study of early radiation cardiotoxicity.^15^

To further elucidate the early effects of contemporary RT, we evaluated changes in cardiac function using 3D echocardiography assessment before, immediately after, and 5 to 9 months after thoracic RT exposure in patients with breast cancer, lung cancer, and mediastinal lymphoma. We hypothesized that detailed 3D echocardiographic phenotyping could reveal dose-dependent, subclinical RT-related left ventricular dysfunction.

## Methods

Patients with breast cancer, lung cancer, or mediastinal lymphoma treated with curative intent photon or proton thoracic RT with or without chemotherapy were enrolled in a prospective longitudinal cohort study between 2015 and 2018 (NCT 02769299). All participants provided written informed consent. The study was approved by the institutional review board.

Transthoracic echocardiography was performed pre-RT, immediately after RT completion, and 5 to 9 months post-RT. The current analysis was a subcohort of 69 patients with quantifiable 3D echocardiograms pre-RT and at least 1 post-RT timepoint. Quantitative 3D echocardiography analyses were performed by a single-blinded observer using the TomTec Imaging Systems platform (Supplementary Methods), with each echocardiogram measured twice by a single observer. The average of the 2 observations was used for analysis.^16^ Intraobserver coefficient of variation was 4.9% for LVEF and ranged between 7.8% and 12.4% for strain measurements (Table E1).

Baseline characteristics were summarized using counts (proportion) for categorical and median (Quartile 1, Quartile 3) for continuous variables. Baseline-adjusted mean changes in 3D echocardiography measures from pre-RT levels were estimated using repeated measures linear regression models via generalized estimating equations. Estimates were additionally determined according to cancer subgroups (patients with breast cancer and patients with lung cancer/mediastinal lymphoma). Patients with lung cancer and mediastinal lymphoma were evaluated in a combined approach given small subgroups and higher relative cardiac radiation dose and also explored individually. In additional exploratory analysis, we evaluated the changes in 3D-echocardiography measures according to subgroups defined based on mean heart dose (MHD; <4 Gy vs ≥4 Gy).

Associations between cardiac radiation exposure and changes in cardiac function were evaluated using repeated measures linear regression via generalized estimating equations. In these models, radiation dose-volume metrics {MHD, percent volume of the heart receiving 5 Gy [V5 Gy (%)] and 30 Gy [V30 Gy (%)]} and changes in 3D echocardiography measures were included as independent variables, whereas changes in measures of cardiac function were included as dependent variables. Models adjusted for age, cancer type, anthracycline exposure, smoking status, hypertension, and diabetes mellitus before RT, study visit, and pre-RT levels of the outcome under consideration.

To account for the possibility of nonrandom nature of missingness in 3D echocardiography measures (Table E2), weighted models incorporating each patient’s inverse probability of inclusion in the analytical subcohort were used (Supplementary Methods).^17^ Given the limited sample size, associations between RT dose and echocardiography measures across individual cancer types were not performed. Statistical analysis was performed using R 4.4.2 (R Foundation for Statistical Computing). A 2-sided significance level of 5% was used to determine statistical significance.

## Results

Baseline characteristics of the 69 patients included in this analysis are summarized in Table 1; characteristics according to cancer type are shown in Table E3. Among the 69 patients, 39 (56.5%) had breast cancer, whereas 13 (18.8%) had lung cancer, and 17 (24.6%) had lymphoma. Overall, the median (quartile 1, quartile 3) MHD was 3 Gy (1, 7); estimates were 1.2 Gy (1.0, 1.9) in patients with breast cancer, 19.4 Gy (11.3, 21.7) in patients with lung cancer, and 6.8 Gy (4.0, 12.5) in patients with lymphoma. Additionally, 32 patients (46.4%) had anthracycline chemotherapy immediately before RT. The number of available 3D-echocardiography measures and distributions at each timepoint is summarized in Table E4.

**Table 1:** Baseline characteristics

Variable	Overall (*N* = 69)*
Age (y)	53 (40, 62)
Female sex	51 (73.9)
Race	
Black/African American	14 (20.3)
White	54 (78.3)
Other	1 (1.4)
Cancer type	
Breast	39 (56.5)
Lung	13 (18.8)
Mediastinal lymphoma	17 (24.6)
Anthracycline chemotherapy as part of current treatment	32 (46.4)
Past anthracycline exposure	6 (8.7)
SBP, mm Hg	123 (119, 134)
DBP, mm Hg	74 (70, 85)
BMI, kg/m^2^	29 (23, 32)
LVEF (2D), %	52 (48, 56)
Current or past smoking	32 (46.4)
Hypertension	20 (29.0)
Diabetes mellitus	6 (8.7)
Statin use	16 (23.2)
ACEI/ARB use	10 (14.5)
Beta-blocker use	12 (17.6)
Primary radiation technique	
3D Conformal	30 (43.5)
IMRT	17 (24.6)
Protons (passive scattering)	5 (7.2)
Protons (scanning)	17 (24.6)
Total radiation dose, Gy	53 (50, 60)
Mean heart dose, Gy	3 (1, 7)
V5 Gy, %	10 (2, 32)
V30 Gy, %	0.7 (0, 7)

*Abbreviations*: ACEI/ARB = angiotensin-converting enzyme inhibitor/angiotensin receptor blocker; BMI = body mass index; DBP = diastolic blood pressure; LVEF = left ventricular ejection fraction; IMRT = intensity modulated radiation therapy; SBP = systolic blood pressure.

V5 and V30 indicate the percent volume of heart receiving 5 Gy and 30 Gy, respectively.

⁎ Includes participants with available analyzable 3D-echocardiogram at baseline and during at least one of the follow-up visits.

**Figure 1:**
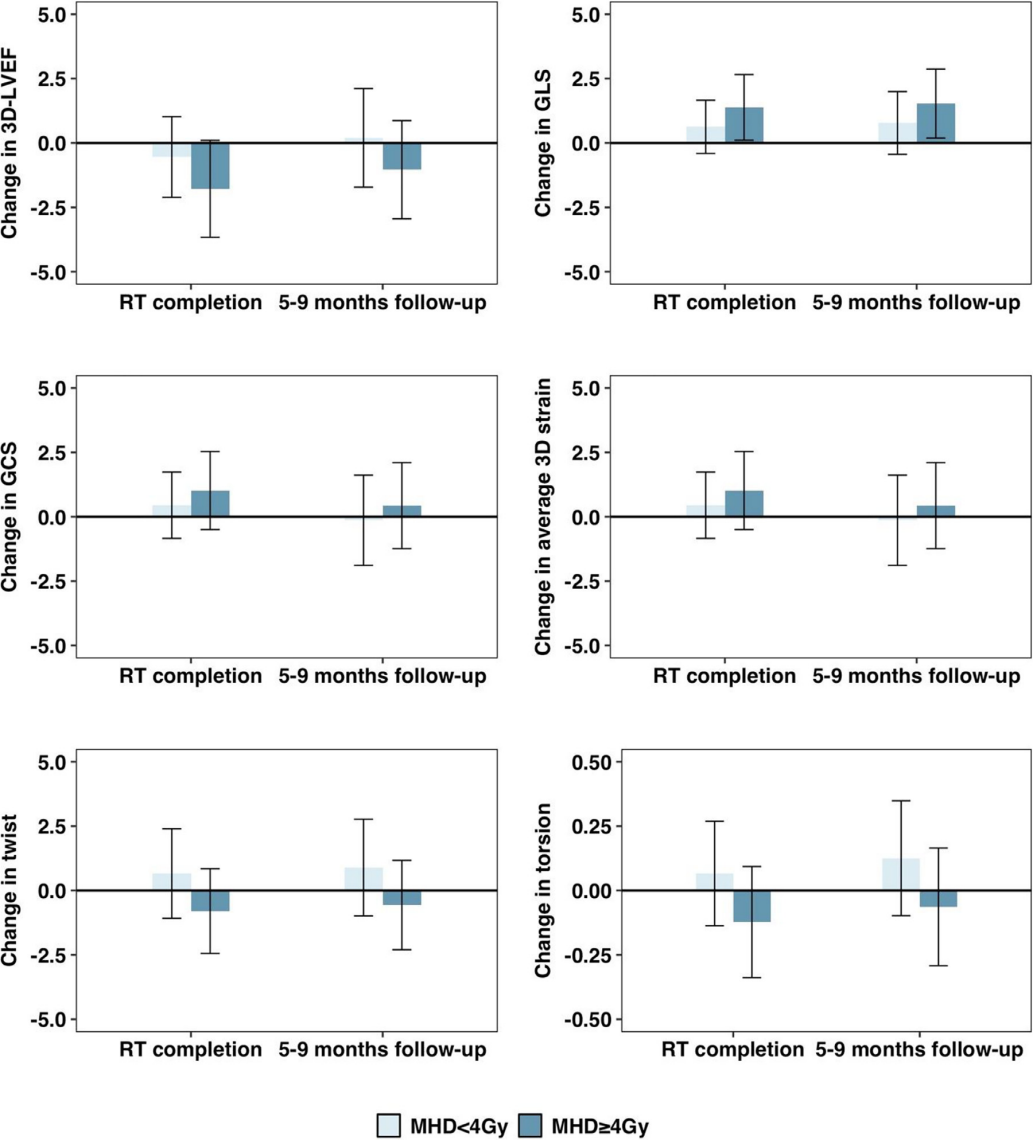
Mean (95% CI) estimated changes in 3D echocardiography measures at post-RT study visits from pre-RT levels according to cancer type. Estimates were determined using repeated measures linear regression estimated via GEE. Models were adjusted for baseline levels of LVEF and study visit number. *Abbreviations*: GCS = global circumferential strain; GEE = generalized estimating equations; GLS = global longitudinal strain; LVEF = left ventricular ejection fraction; RT = radiation therapy.

Estimates of mean changes in 3D echocardiography measures from pre-RT levels in the overall cohort are presented in Table 2. There were no significant changes in 3D echocardiography measures following RT. There were, however, differences in the pattern of changes in these measures in cancer type-stratified analysis. Although there were no significant changes in 3D echocardiography measures in patients with breast cancer following RT, a worsening in 3D LVEF, global longitudinal strain (GLS), and average 3D strain was observed from pre-RT to RT completion in patients with lung cancer/lymphoma. On average, LVEF decreased by 2.1% (95% CI, –3.9%, –0.2%, *P* = .03), GLS worsened by 1.6 (95% CI, 0.2, 3.1, *P* = .03) and average 3D strain decreased by 2.0 (95% CI, 0.1, 3.9, *P* = .04). The worsening in GLS in patients with lung cancer/lymphoma persisted at the 5 to 9 months follow-up timepoint (Fig. 1, Table 3). There were no statistically significant changes in twist or torsion after RT observed within both subgroups. In exploratory analysis with lung cancer and lymphoma subgroups evaluated individually, the strength of these associations was attenuated, likely given small sample size, but were consistent for GLS, GCS, and 3D strain in patients with lymphoma (Table E5). In additional exploratory analysis, greater worsening in 3D LVEF and strain was observed in patients with MHD exposure ≥4 Gy (Fig. E1). In patients with MHD exposure ≥4 Gy, there was a statistically significant worsening in GLS observed at both RT completion (95% CI, 0.1, 2.7, *P* = .03) and at the 5 to 9 months follow-up timepoints (95% CI, 0.2, 2.9, *P* = .02) (Table E6**)**.

**Table 2:** Baseline-adjusted mean (95% CI) estimates of change in 3D echocardiography measures at post-RT timepoints from pre-RT levels in the overall cohort

	Change from pre-RT			
	RT completion		5-9 mo follow-up	
3D-echocardiography measure	Mean (95% CI)	*P* value	Mean (95% CI)	*P* value
LVEF	–0.9 (–2.6, 0.8)	.30	0.2 (–1.8, 2.2)	.86
GLS	0.8 (–0.2, 1.8)	.13	0.8 (–0.4, 2.1)	.21
GCS	0.5 (–0.8, 1.8)	.47	–0.4 (–2.3, 1.5)	.68
Average 3D strain	1.1 (–0.4, 2.5)	.15	0.5 (–1.5, 2.4)	.64
Twist	0.6 (–1.2, 2.4)	.50	0.9 (–0.9, 2.7)	.32
Torsion	0.1 (–0.2, 0.3)	.59	0.1 (–0.1, 0.3)	.27

*Abbreviations*: CI = confidence interval; GCS = global circumferential strain; GEE = generalized estimating equations; GLS = global longitudinal strain; LVEF = left ventricular ejection fraction; MHD = mean heart dose; RT = radiation therapy.

Estimates were determined using repeated measures linear regression estimated via GEE. Models included study visit number and baseline levels of the 3D-echocardiography measure under consideration.

**Table 3:** Baseline-adjusted mean (95% CI) estimates of change in 3D echocardiography measures at post-RT timepoints from pre-RT levels in the overall cohort

	Breast cancer				Lymphoma/lung cancer			
	RT completion		5-9 mo follow-up		RT completion		5-9 mo follow-up	
3D-echocardiography measure	Mean (95% CI)	*P* value	Mean (95% CI)	*P* value	Mean (95% CI)	*P* value	Mean (95% CI)	*P* value
LVEF	–0.2 (–2.1, 1.6)	.81	0.7 (–1.2, 2.7)	.45	–2.1 (–3.9, –0.2)	.03	–1.1 (–3.4, 1.2)	.35
GLS	0.3 (–0.7, 1.3)	.50	0.4 (–0.8, 1.6)	.50	1.6 (0.2, 3.1)	.03	1.7 (0.1, 3.3)	.04
GCS	0.1 (–1.4, 1.5)	.91	–0.7 (–2.6, 1.2)	.45	1.2 (–0.3, 2.7)	.11	0.4 (–1.6, 2.4)	.70
Average 3D strain	0.5 (–0.9, 2.0)	.46	0.02 (–1.8, 1.9)	.98	2.0 (0.1, 3.9)	.04	1.5 (–0.9, 3.8)	.23
Twist	1.1 (–0.9, 3.1)	.30	1.3 (–0.6, 3.2)	.19	–0.2 (–1.9, 1.5)	.83	0.02 (–1.8, 1.8)	.98
Torsion	0.1 (–0.1, 0.3)	.36	0.2 (–0.1, 0.4)	.17	–0.04 (–0.1, 0.3)	.72	0.02 (–0.2, 0.2)	.86

*Abbreviations*: CI = confidence interval; GCS = global circumferential strain; GEE = generalized estimating equations; GLS = global longitudinal strain; LVEF = left ventricular ejection fraction; MHD = mean heart dose; RT = radiation therapy.

Estimates were determined using repeated measures linear regression estimated via GEE. Models included study visit number and baseline levels of the 3D-echocardiography measure under consideration.

Multivariable associations between radiation dose-volume metrics and changes in 3D echocardiography measures from pre-RT levels are presented in Table 4. There were no statistically significant associations between MHD, V5, and V30 and early changes in 3D echocardiography measures across the entire cohort.

**Table 4:** Associations between radiation dose-volume metrics and changes in 3D echocardiography measures at post-RT timepoints from pre-RT levels

	MHD*		V5 Gy, %†		V30 Gy, %†	
3D-echocardiography measure	Mean difference (95% CI)	*P* value	Mean difference (95% CI)	*P* value	Mean difference (95% CI)	*P* value
LVEF	0.1 (–0.1, 0.4)	.21	0.5 (–0.2, 1.2)	.19	0.5 (–0.8, 1.7)	.48
GLS	–0.1 (–0.3, 0.2)	.53	–0.3 (–1.0, 0.4)	.37	–0.1 (–1.2, 1.0)	.88
GCS	–0.2 (–0.4, 0.1)	.09	–0.4 (–1.0, 0.1)	.11	–0.6 (–1.7, 0.4)	.24
Average 3D strain	–0.1 (–0.4, 0.1)	.24	–0.4 (–1.1, 0.2)	.18	–0.4 (–1.6, 0.7)	.46
Twist	0.1 (–0.1, 0.2)	.36	0.4 (–0.1, 1.0)	.10	0.2 (–0.9, 1.4)	.70
Torsion	0.01 (–0.01, 0.03)	.49	0.04 (–0.02, 0.11)	019	0.02 (–0.14, 0.18)	.82

*Abbreviations*: CI = confidence interval; GCS = global circumferential strain; GEE = generalized estimating equations; GLS = global longitudinal strain; LVEF = left ventricular ejection fraction; MHD = mean heart dose; RT = radiation therapy.

V5 and V30 indicate the percent volume of heart receiving 5 Gy and 30 Gy, respectively.

Associations were modeled using repeated measures linear regression estimated via GEE. Models adjusted for age, cancer type, anthracycline exposure before RT (yes vs no), current or past smoking, hypertension, diabetes mellitus, study visit number, and baseline levels of the outcome under consideration

⁎ Estimates should be interpreted as the mean difference in the outcome under consideration for each 1 Gy increase.

† Estimates should be interpreted as the mean difference in the outcome under consideration for each 10% increase.

## Discussion

In this prospective longitudinal cohort study of 69 patients with breast cancer, lung cancer, or mediastinal lymphoma, changes in 3D echocardiography measures of cardiac function following thoracic RT and their associations with cardiac radiation dose-volume metrics were evaluated. There were 3 main findings: (1) there were no significant changes in 3D echocardiography measures in patients with breast cancer; (2) there was a modest, but statistically significant worsening in 3D LVEF, GLS, and average 3D strain from pre-RT to RT completion in patients with lung cancer/lymphoma, with a sustained worsening of 3D GLS at the 5 to 9 months timepoint; and (3) there were no significant associations between MHD, V5, and V30 with changes in 3D echocardiography-derived measures of cardiac function across the entire cohort.

This study adds to a limited, but growing body of research that explores the use of a comprehensive list of 3D echocardiographic parameters in evaluating subclinical changes in cardiac systolic function following thoracic RT. There are few studies that have performed dedicated analysis on the subclinical, acute cardiotoxic effects of RT in patients with lung cancer or lymphoma. We determined a sustained worsening of 3D LV GLS 5 to 9 months following RT in patients with lung cancer/lymphoma. We believe the changes observed within patients with lung cancer/lymphoma are likely in part related to RT exposure, given the pattern of greater worsening in 3D LVEF and strain observed in patients with MHD exposure ≥4 Gy and the included statistical analyses controlled for several potential confounders, including anthracycline exposure which has been shown to affect left ventricular function.

However, across the entire cohort, we did not detect statistically significant associations between MHD, V5, and V30 and early changes in cardiac function. There are several possible reasons for the lack of association between radiation dose-volume metrics and changes in cardiac function in this study. The sample size was small and the lack of statistical power could have limited the ability to detect significant associations. Additionally, one cannot exclude the possibility of more complex, nonlinear relationships between radiation dose-volume metrics and changes in cardiac function. It is also possible that global metrics, such as MHD, that quantify radiation exposure to the heart do not adequately reflect, and thus are not sensitive enough, to predict subclinical and acute, early changes in global measures of cardiac function. There has been growing evidence to suggest that RT doses to cardiac substructures, such as the coronary arteries, could be more sensitive in predicting abnormalities in cardiac function, particularly as it relates to patients with lung cancer.^18^^,^^19^ Moreover, evaluation of segmental changes in cardiac function may be of value.

The study has limitations. The study was performed in a single quaternary care academic medical center, which could impact generalizability. Our sample size was small, limiting our analysis in fully understanding the associations between radiation dose-volume metrics and cardiac function in individual cancer types. Furthermore, we could not evaluate more complex and possibly nonlinear relationships between radiation dose-volume metrics and changes in cardiac function because of limited statistical power*.* The lack of data on radiation dose-volume metrics specific to cardiac substructures is another limitation of our study. Finally, we lacked complete 3D echocardiography data. 3D echocardiographic analysis remains especially sensitive to image quality as this technique does not rely on geometric assumptions of the LV as employed with 2D echocardiography and therefore deterioration of image quality during follow up is more likely to affect 3D images.^20^ We attempted to address this issue of missing 3D data by using a mean score estimation method but acknowledge that this methodology may not fully compensate for the missing data and affect the study’s overall validity. However, we also note strengths, including a clinically relevant question, detailed echocardiographic phenotyping before and early after RT, and comprehensive, rigorous statistical modeling approaches.

## Conclusion

Early abnormalities in cardiac function as measured by 3D echocardiography measures are observed in patients following RT. Further research is needed to ascertain the causal relationship between RT and changes in cardiac function as well as the long-term impact of these changes on clinical outcomes.

## Disclosures

The authors declare that they have no known competing financial interests or personal relationships that could have appeared to influence the work reported in this paper.

## Appendix Supplementary materials

ADVANCESRADONC-D-24-00265_R2_Supplementary Material Image, application 1

## Footnotes

Supplementary material associated with this article can be found in the online version at doi:10.1016/j.adro.2025.101786.
