# Supplementary material for: 3D Echocardiographic Phenotyping of Left Ventricular Mechanics and Function With Contemporary Radiation Therapy
Source: Adv Radiat Oncol. 2025 Apr 16;10(7):101786. doi: 10.1016/j.adro.2025.101786 (PMC12155620; doi:10.1016/j.adro.2025.101786)
Supplement: ADVANCESRADONC-D-24-00265_R2_Supplementary Material [file mmc1.docx]

**Supplementary material**

**Supplementary Methods**

Given that the 3D-echocardiography outcomes were available in a subsample of participants enrolled in the Cardiotoxicity of Radiation Therapy cohort, the mean score method was used. The mean score method has been shown to provide more efficient estimation when the true outcome of interest is only available in a subsample but surrogate (auxiliary) data are available in the whole sample (Chen et al, Can J Statistics 2004;32:359-372; Pepe et al, Journal of Statistical Planning and Inference 1994;42:137-160). Furthermore, this method can reduce potential biases related to a possible non-random nature of missingness in 3D-echocardiography outcomes (see Supplementary Table 2) by incorporating the auxiliary outcome and other covariate information from the whole sample. The following steps were implemented to incorporate this method:

Step 1: In the whole sample, fit a binomial logistic regression model for inclusion in the analytic subsample as a dependent variable using an auxiliary outcome and covariates as independent variables. We used 2D-LVEF as the auxiliary outcome while the list of covariates included age, sex, cancer type, anthracycline treatment, systolic blood pressure, BMI, smoking, hypertension, diabetes, statin use, ACEI/ARB use, beta-blocker use and mean heart dose.

Step 2: Calculate probability weights for each individual participant based on the model in step 1.

Step 3: Perform primary analysis in the subsample of participants with available 3D-echocardiography outcomes. Here, the inverse of the probability weights calculated in step 2 will be included as sampling weights in the different regression models.

Supplementary Table 1: Intra-reader reproducibility analysis for 3D-echocardiography measures

| 3D-Echocardiography measure | Coefficient of variation (%) | Bland-Altman mean difference (SD) |
| --- | --- | --- |
| LVEF | 4.9 | 2.1 (3.4) |
| GLS | 12.4 | 1.8 (3.2) |
| GCS | 8.8 | 1.4 (3.3) |
| Average strain | 7.8 | 1.5 (3.4) |
| Twist | 10.5 | 0.2 (1.5) |
| Torsion | 11.5 | 0 (0.2) |
| Reproducibility analysis was performed using pairs of measurements from a single observer on 40 randomly selected echocardiograms | | |

Supplementary Table 2: Comparison of baseline characteristics of included versus excluded participants

| Variable | Excluded (n=67) | Included* (n=69) | P-value |
| --- | --- | --- | --- |
| Age, years | 55 (44, 63) | 53 (40, 62) | 0.26 |
| Female sex | 56 (83.6) | 51 (73.9) | 0.24 |
| Race |  |  | 0.51 |
| Black/African American | 11 (16.4) | 14 (20.3) |  |
| White | 53 (79.1) | 54 (78.3) |  |
| Other | 3 (4.5) | 1 (1.4) |  |
| Disease site |  |  | 0.02 |
| Breast | 45 (67.2) | 39 (56.5) |  |
| Lung | 17 (25.4) | 13 (18.8) |  |
| Mediastinal lymphoma | 5 (7.5) | 17 (24.6) |  |
| Anthracycline chemotherapy as part of current treatment | 18 (26.9) | 32 (46.4) | 0.03 |
| Past anthracycline exposure | 5 (7.5) | 6 (8.7) | >0.99 |
| SBP, mmHg | 129 (114, 142) | 123 (119, 134) | 0.34 |
| DBP, mmHg | 75 (70, 84) | 74 (70, 85) | 0.61 |
| BMI, kg/m^2^ | 28 (23, 33) | 29 (23, 32) | 0.76 |
| LVEF (2D), % | 52 (50, 56) | 53 (50, 56) | 0.78 |
| Current or past smoking | 31 (46.3) | 32 (46.4) | >0.99 |
| Hypertension | 24 (35.8) | 20 (29.0) | 0.50 |
| Diabetes mellitus | 9 (13.4) | 6 (8.7) | 0.54 |
| Statin use | 14 (20.9) | 16 (23.2) | 0.91 |
| ACEI/ARB use | 16 (23.9) | 10 (14.5) | 0.24 |
| Beta-blocker use | 17 (25.4) | 12 (17.4) | 0.35 |
| Mean heart dose, Gy | 265 (119, 674) | 251 (115, 687) | 0.95 |
| V5 Gy, % | 10 (2, 24) | 9 (2, 31) | 0.76 |
| V30 Gy, % | 0.9 (0, 7) | 0.7 (0, 7.2) | 0.72 |
| * Includes participants with analyzable 3D-echocardiogram at baseline (i.e., pre-RT) and during at least one of the follow-up visits | | |  |

Supplementary Table 3: Baseline characteristics according to cancer type (breast cancer and lung cancer/mediastinal lymphoma

| Variable | Breast cancer (n=39) | Lung cancer/mediastinal lymphoma (n=30) | P-value |
| --- | --- | --- | --- |
| Age, years | 53 (43, 61) | 51 (27, 64) | 0.19 |
| Female sex | 39 (100) | 12 (40.0) | <0.001 |
| Race |  |  |  |
| Black/African American | 11 (28.2) | 3 (10.0) | 0.10 |
| White | 28 (71.8) | 26 (86.7) |  |
| Other | 0 (0) | 1 (3.3) |  |
| Anthracycline chemotherapy as part of current treatment | 20 (51.3) | 12 (40.0) | 0.49 |
| Past anthracycline exposure | 1 (2.6) | 5 (16.7) | 0.10 |
| SBP, mmHg | 123 (116, 138) | 123 (120, 132) | 0.96 |
| DBP, mmHg | 74 (69, 86) | 75 (70, 80) | 0.50 |
| BMI, kg/m^2^ | 29 (24, 32) | 29 (24, 31) | 0.98 |
| LVEF (2D), % | 53 (50, 57) | 52 (50, 54) | 0.17 |
| Current or past smoking | 17 (43.6) | 15 (50.0) | 0.78 |
| Hypertension | 13 (33.3) | 7 (23.3) | 0.52 |
| Diabetes mellitus | 5 (12.8) | 1 (3.3) | 0.34 |
| Statin use | 10 (25.6) | 6 (20.0) | 0.79 |
| ACEI/ARB use | 4 (10.3) | 6 (20..0) | 0.43 |
| Beta-blocker use | 8 (20.5) | 4 (13.3) | 0.65 |
| Primary radiation technique |  |  | <0.001 |
| 3D Conformal | 28 (71.8) | 2 (6.7) |  |
| IMRT | 4 (10.3) | 13 (43.3) |  |
| Protons (passive scattering) | 3 (7.7) | 2 (6.7) |  |
| Protons (scanning) | 4 (10.3) | 13 (43.3) |  |
| Total radiation dose, Gy | 53 (52, 60) | 40 (31, 65) | 0.04 |
| Mean heart dose, Gy | 1.2 (1, 1.9) | 10.9 (4,4, 19.2) | <0.001 |
| V5 Gy, % | 2.2 (1.2, 6.6) | 40.0 (23.0, 67.0) | <0.001 |
| V30 Gy, % | 0.1 (0, 0.7) | 13.9 (2.8, 28.7) | <0.001 |

Supplementary Table 4: Summary of available 3D-echocardiography measures according to study timepoint

| **3D-echocardiography measure** | **Number of available values** | | | **Median (Q1, Q3)** | | |
| --- | --- | --- | --- | --- | --- | --- |
|  | **Pre-radiation** | **Radiation completion** | **5-9 months follow-up** | **Pre-radiation** | **Radiation completion** | **5-9 months follow-up** |
| LVEF | 69 | 60 | 43 | 56 (53,60) | 56 (52,59) | 57 (54,59) |
| GLS | 69 | 60 | 43 | -22 (-24,-19) | -20 (-24,-17) | -21 (-23,-19) |
| GCS | 69 | 60 | 43 | -28 (-31,-25) | -27 (-29,-24) | -28 (-31,-25) |
| Average 3D strain | 69 | 60 | 43 | -33 (-37,-30) | -32 (-35,-29) | -34 (-36,-30) |
| Twist | 69 | 60 | 43 | 7 (4,10) | 7 (3,13) | 7 (4,11) |
| Torsion | 69 | 60 | 43 | 0.8 (0.5,1.2) | 0.8 (0.5,1.6) | 0.9 (0.5,1.4) |

Supplementary Table 5: Baseline-adjusted mean (95% CI) estimates of change in 3D echocardiography measures at post-RT timepoints from pre-RT levels in lung cancer and mediastinal lymphoma

| 3D-echocardiography measure | Lung cancer (n=13) | | Mediastinal lymphoma (n=17) | |
| --- | --- | --- | --- | --- |
|  | RT completion | 5-9 months follow-up | RT completion | 5-9 months follow-up |
| LVEF | -1.9 (-4.4, 0.5) | -1.2 (-3.7, 1.2) | -2.0 (-3.9, -0.02) | -1.3 (-3.7, 1.1) |
| GLS | 1.3 (-0.6, 3.2) | 1.5 (-0.4, 3.4) | 1.7 (0.4, 3.0) | 1.9 (0.3, 3.5) |
| GCS | 0.8 (-1.2, 2.8) | 0.2 (-1.9, 2.4) | 1.5 (0.02, 3.0) | 0.9 (-0.9, 2.8) |
| Average 3D strain | 1.5 (-1.2, 4.2) | 1.2 (-1.5, 3.9) | 2.2 (0.5, 3.9) | 1.9 (-0.2, 4.0) |
| Twist | -0.4 (-2.4, 1.6) | -0.2 (-2.2, 1.8) | -0.7 (-2.4, 1.6) | -0.5 (-2.5, 1.4) |
| Torsion | -0.04 (-0.30, 0.21) | 0.01 (-2.16, 1.81) | -0.11 (-0.33, 0.10) | -0.06 (-0.31, 0.18) |
| Estimates were determined using repeated measures linear regression estimated via GEE. Models included study visit number and baseline levels of the 3D-echocardiography measure under consideration. *Abbreviations*: LVEF = left ventricular ejection fraction; GLS = global longitudinal strain; GCS = global circumferential strain; MHD = mean heart dose; CI = confidence interval | | | | |

Supplementary Table 6: Baseline-adjusted mean (95% CI) estimates of change in 3D echocardiography measures at post-RT timepoints from pre-RT levels according to MHD Cut-point of 4 Gy

| 3D-echocardiography measure | MHD<4 Gy | | | | MHD≥4 Gy | | | |
| --- | --- | --- | --- | --- | --- | --- | --- | --- |
|  | RT completion | | 5-9 months follow-up | | RT completion | | 5-9 months follow-up | |
|  | Mean (95% CI) | P-value | Mean (95% CI) | P-value | Mean (95% CI) | P-value | Mean (95% CI) | P-value |
| LVEF | -0.5 (-2.1, 1.0) | 0.49 | 0.2 (-1.7, 2.1) | 0.84 | -1.8 (-3.7, 0.1) | 0.06 | -1.0 (-2.9, 0.9) | 0.28 |
| GLS | 0.6 (-0.4, 1.7) | 0.23 | 0.8 (-0.4, 2.0) | 0.21 | 1.4 (0.1, 2.7) | 0.03 | 1.5 (0.2, 2.9) | 0.02 |
| GCS | 0.4 (-0.8, 1.7) | 0.49 | -0.1 (-1.9, 1.6) | 0.88 | 1.0 (-0.5, 2.5) | 0.18 | 0.4 (-1.2, 2.1) | 0.61 |
| Average 3D strain | 0.8 (-0.5, 2.1) | 0.24 | 0.4 (-1.2, 2.1) | 0.59 | 1.7 (0.2, 3.6) | 0.07 | 1.4 (-0.6, 3.3) | 0.16 |
| Twist | 0.7 (-1.1, 2.4) | 0.45 | 0.9 (-1.0, 2.8) | 0.35 | -0.8 (-2.4, 0.8) | 0.34 | -0.6 (-2.3, 1.2) | 0.52 |
| Torsion | 0.1 (-0.1, 0.3) | 0.52 | 0.1 (-0.1, 0.3) | 0.27 | -0.1 (-0.3, 0.1) | 0.26 | -0.1 (-0.3, 0.2) | 0.58 |
| Estimates were determined using repeated measures linear regression estimated via GEE. Models included study visit number and baseline levels of the 3D-echocardiography measure under consideration. *Abbreviations*: LVEF = left ventricular ejection fraction; GLS = global longitudinal strain; GCS = global circumferential strain; MHD = mean heart dose; CI = confidence interval | | | | | | | | |


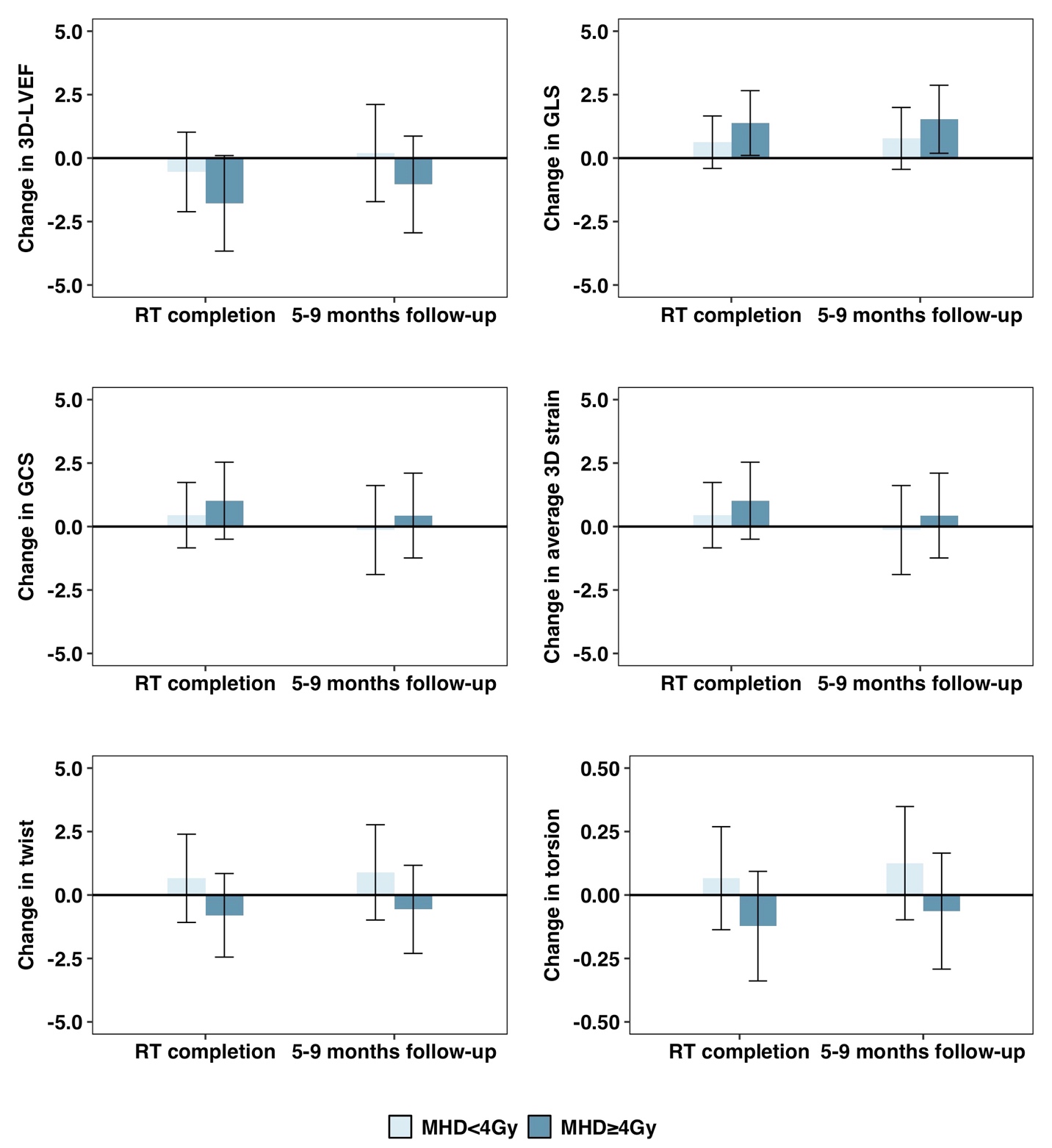


Supplementary Figure 1: Changes in 3D-echocardiography measures of left ventricular function according to mean heart dose cut-point of 4 Gy
